# IL-1β Induced Cytokine Expression by Spinal Astrocytes Can Play a Role in the Maintenance of Chronic Inflammatory Pain

**DOI:** 10.3389/fphys.2020.543331

**Published:** 2020-11-16

**Authors:** Andrea Gajtkó, Erzsébet Bakk, Krisztina Hegedűs, László Ducza, Krisztina Holló

**Affiliations:** Department of Anatomy, Histology and Embryology, Faculty of Medicine, University of Debrecen, Debrecen, Hungary

**Keywords:** IL-1beta, astrocyte, spinal cord, chronic pain, inflammatory cytokines and chemokines

## Abstract

It is now widely accepted that the glial cells of the central nervous system (CNS) are key players in many processes, especially when they are activated via neuron-glia or glia-glia interactions. In turn, many of the glia-derived pro-inflammatory cytokines contribute to central sensitization during inflammation or nerve injury-evoked pathological pain conditions. The prototype of pro-inflammatory cytokines is interleukin-1beta (IL-1β) which has widespread functions in inflammatory processes. Our earlier findings showed that in the spinal cord (besides neurons) astrocytes express the ligand binding interleukin-1 receptor type 1 (IL-1R1) subunit of the IL-1 receptor in the spinal dorsal horn in the chronic phase of inflammatory pain. Interestingly, spinal astrocytes are also the main source of the IL-1β itself which in turn acts on its neuronal and astrocytic IL-1R1 leading to cell-type specific responses. In the initial experiments we measured the IL-1β concentration in the spinal cord of C57BL/6 mice during the course of complete Freund adjuvant (CFA)-induced inflammatory pain and observed a peak of IL-1β level at the time of highest mechanical sensitivity. In order to further study astrocytic activation, primary astrocyte cultures from spinal cords of C57BL/6 wild type and IL-1R1 deficient mice were exposed to IL-1β in concentrations corresponding to the spinal levels in the CFA-induced pain model. By using cytokine array method we observed significant increase in the expressional level of three cytokines: interleukin-6 (IL-6), granulocyte-macrophage colony stimulating factor (GM-CSF) and chemokine (C-C motif) ligand 5 (CCL5 or RANTES). We also observed that the secretion of the three cytokines is mediated by the NFkB signaling pathway. Our data completes the picture of the IL-1β-triggered cytokine cascade in spinal astrocytes, which may lead to enhanced activation of the local cells (neurons and glia as well) and can lead to the prolonged maintenance of chronic pain. All these cytokines and the NFkB pathway can be possible targets of pain therapy.

## Introduction

Astrocytes are the most abundant glial cells in the CNS, they are responsible for many functions as supporting cells in the central nervous system (CNS) e.g., maintenance of the ionic milieu, induction of the blood brain barrier, removal of excess neurotransmitters etc. (Verkhratsky and Nedergaard, 2018). However, it is clear now, that astrocytes are not merely supporting cells in the CNS, but they are capable to modulate neuronal excitability (Suter et al., 2007; Milligan and Watkins, 2009; Kuner, 2010; Ji et al., 2019) and in this way contribute to the onset and maintenance of numerous CNS pathologies including chronic pain (Gao and Ji, 2010; Chiang et al., 2012; Liddelow and Barres, 2015). One possible way of modulating neuronal activity is the astroglial production of cytokines and chemokines which can act on their neuronal receptors (Zhang and An, 2007) thus contribute to neuron-glia interactions. Such enhanced cytokine expression was observed in neuropathic and inflammatory pain as well (Calvo et al., 2012; Jayaraj et al., 2019). Some of these cytokines and chemokines are also means of glia-glia communication as their receptors are expressed by glial cells (Conti et al., 2008; Trettel et al., 2019).

In this study we focused on the role of interleukin-1β (IL-1β) which is the prototype of pro-inflammatory cytokines, it is a regulator of many immunological functions (Sims and Smith, 2010). It was shown to be involved in the pathomechanism of several inflammatory disorders which are also associated with pain (Dinarello et al., 2012). In the CNS its receptor (IL-1R1) was found to be expressed by neurons (Niederberger et al., 2007; Cao and Zhang, 2008; Zhu et al., 2008) and glial cells (Wang et al., 2006; Zhu et al., 2008; Liao et al., 2011; Gruber-Schoffnegger et al., 2013). It has been reported that IL-1β can induce astrocytic activation and astrogliosis (Herx and Yong, 2001). Our earlier findings (Holló et al., 2017) also suggest that during inflammatory pain IL-1β can act on spinal neurons and astrocytes. It has been revealed that IL-1β induce cell type-specific response in the cells of the CNS due to a neuron specific isoform of the IL-1 receptor accessory protein (IL-1RAcP), which is a required receptor partner in IL-1 signaling (Huang et al., 2011). In nerve cells IL-1β modulates neuronal excitability by e.g., potentiation of NMDA-mediated intracellular calcium signaling (Viviani et al., 2003; Cao and Zhang, 2008), while IL-1β activated astrocytes produce a cascade of inflammatory mediators which can further enhance and possibly prolong neuroinflammation-induced chronic pain (Zhang and An, 2007).

In this study we intended to identify those cytokines and chemokines which are secreted by IL-1β-stimulated spinal astrocytes. We also intended to investigate the activation of the NF-kB signaling pathway which is associated with astrocyte-specific IL-1β signaling.

## Materials and Methods

### Animals

The study protocol was reviewed and approved by the recommendations of the Animal Care Committee of the University of Debrecen, Hungary according to national laws and European Union regulations [European Communities Council Directive of 24 November 1986 (86/609/EEC)], and was properly conducted under the supervision of the University’s Guidelines for Animal Experimentation. All animals were kept under standard conditions with chow and water *ad libitum*. The experiments were performed on male C57BL/6 mice (Gödöllő, Hungary). The animals were divided into experimental groups. Experimental group 1 (6 control mice) and experimental group 2 (21 CFA treated animals). In animals within the treated group chronic inflammation was induced by intraplantar injection of 50 μl 1:1 mixture of physiological saline solution and complete Freund-adjuvant (CFA) (Sigma, St Louis, United States) into the right hindpaw of mice according to the method described earlier (Hylden et al., 1989).

### Nociceptive Behavioral Test

Control and CFA-treated animals were tested for paw withdrawal responses to noxious mechanical stimuli. Mechanical sensitivity of the animals was detected by a modified von Frey test (Dynamic Plantar Aesthesiometer, Ugo Basile, Gemonio, Italy). Animals were placed into a cage with acrylic sidewalls and mesh floor. After 15 min of habituation, a flexible, von Frey-type filament (diameter = 0.5 mm) exerted increasing force on the plantar surface of the hindpaw, until the animal withdrew it. The mechanical withdrawal threshold (MWT) for both hind paws were measured before CFA injection and the tests were repeated daily after CFA injection. The MWT was detected automatically. The test was repeated five times for each paw with 2 min intervals alternating between the right and the left paw. From the experimental data mean value and standard error of mean (SEM) were calculated. Statistical differences among the data were calculated according to the One Way ANOVA test.

### IL-1β Quantitative ELISA

Mouse IL-1β/IL-1F2 Quantikine ELISA kit (RnD Systems, Minneapolis, United States, cat. no. MLB00C) was applied for the measurement of total IL-1β amount in spinal cord tissue homogenates. Briefly, control animals (*n* = 3) and CFA-treated animals (*n* = 3/day) on experimental days 1–5 were sacrificed (after measuring the mechanical pain sensitivity levels), the spinal cord was dissected and the dorsal horn of the L4–L5 segments was removed, the treated (right side) and the non-treated (left side) of the tissue was handled separately. After measuring their weight, the tissue samples were mechanically homogenized in ice cold RIPA buffer supplemented with protease inhibitors (Pierce Protease Inhibitor Mini tablet, Thermo Scientific, Rockford, United States). After 20 min. of gentle rocking on ice the samples were centrifuged (10 min, 15000 rpm, at 4°C) to remove insoluble tissue debris. 50 μl of supernatant was used in triplicates to determine the IL-1β content of the tissue homogenates. Then the experiments were performed according to the instructions by the manufacturer. Finally, the IL-1β content was calculated for 1 mg of spinal cord tissue.

### Primary Astrocyte Cultures

For the production of spinal cord astrocyte cultures we basically followed the procedure already described (Hegyi et al., 2018) with slight modifications. Briefly, the whole spinal cord was removed from 2 to 4 day old C57BL/6 and IL-1 receptor type-1 (IL-1R1) deficient/(B6.129S7-Il1r1 tm1/mx,/J stock #:003245) created by Labow et al. (1997) and purchased from Jackson Laboratories (Bar Harbor, ME, United States)/pups after decapitation and placed into ice-cold dissecting buffer (136 mM NaCl, 5.2 mM KCl, 0.64 mM Na_2_HPO4, 0.22 mM KH_2_PO_4_, 16.6 mM glucose, 22 mM sucrose, 10 mM HEPES supplemented with 0.06 U/ml penicillin and 0.06 U/ml streptomycin). The isolated spinal cords were carefully cleaned to remove meninges then placed into fresh dissecting solution containing 0.025 g/ml bovine trypsin (Sigma, St Louis, United States) and were incubated at 37°C for 30 min. Then the solution was replaced by Minimum Essential Medium (MEM, Gibco, Life Technologies Ltd., Parsley, United Kingdom) supplemented with 10% Fetal Bovine Serum (FBS, Hyclone, GE Healthcare Bio-Sciences, Pittsburgh, United States). After 5 min. incubation at room temperature the tissue pieces were gently suspended by a Pasteur pipette and the cell suspension was filtered through a nylon mesh (Cell Strainer, Sigma, pore size: 100 μm), the cell number was identified and the suspension was diluted to a cell density of 1 × 10^6^/ml, the cells were placed into 24 well tissue culture plates (0.5 ml/well). The cell cultures were kept at 37°C in a 5% CO_2_ atmosphere, the medium was replaced the following day and every second day thereafter.

### 3-[4,5-Dimethylthiazole-2-yl]-2,5-Diphenyltetrazolium Bromide (MTT) Assay

Astrocyte cultures prepared from spinal cords of C57/BL6 wild type and IL-1R1-deficient mice were used to test the mitochondrial activity of the MTT assay. Briefly, after 24 h stimulation with increasing concentrations (0.1, 1, 10, 25, and 100 pg/ml) of recombinant murine IL-1β protein (cat. no. 211-11B, PeproTech, Rocky Hill, United States) the culture media was replaced by PBS containing 0.1 mg/ml MTT. After 4 h incubation the supernatants were removed and the formazan product was dissolved in isopropyl alcohol. Finally, the absorbance was measured at λ = 570 nm by microplate reader (Titertek Uniscan, Flow Laboratories, Helsinki, Finland) in duplicates. Absorbance values were averaged and statistical differences were calculated by ANOVA statistical probe.

### Proteome Profiler Assay

The Proteome Profiler Mouse Cytokine Array Kit, Panel A (R&D Systems, cat. no. ARY006) was utilized for the parallel determination of the relative levels of selected mouse cytokines and chemokines produced by the primary astrocyte cultures. Prior to the assay, cells were stimulated by 10 pg/ml recombinant murine IL-1β protein (PeproTech) for 24 h. Then the supernatants were pooled from 12 well non-treated, control and 12 well IL-1β-stimulated cultures. After centrifugation (10 min, 800 rpm at 4°C) 1 ml of the supernatants were mixed with Array Buffer and the Antibody Detection Coctail and were added to the nitrocellulose membranes pre-coated with the capture antibodies. Then we followed the instructions by the manufacturer. The signal was developed by DuoLux Chemiluminescent Substrate Kit (Vector Laboratories, Burlingame, United States, cat. no. SK-6604) and the image was captured by FluorChem E (Protein Simple, San Jose, United States).

The developed membranes were analyzed by the Image-J software. First, pixel densities were determined for each spot on the membranes, then the background signal was subtracted from each value. Finally, we compared the corresponding signals on different arrays to determine the relative change (fold change) in cytokine levels between samples. Statistical differences among the data were calculated according to the ANOVA test, the difference between groups was considered significant if *p* ≤ 0.05.

### Immunohistochemistry

Immunohistochemistry was performed on astrocyte cultures which were kept on coverslips placed into 24 well culture dishes. After 7–10 days of culturing the coverslips were removed and the cells were fixed with 4% paraformaldehyde (15 min.). Then the cells were washed in PBS containing 100 mM glycine followed by 10 min. incubation in PBS. Aspecific labeling was blocked by PBS containing 10% normal serum for 50 min. Then the cells were incubated with the primary antibodies (anti-IL-6, anti-GM-CSF, anti-CCL5,/PeproTech, produced in rabbit); anti-NF-κB p65/Thermo Fisher-Invitrogen, Waltham, United States, produced in rabbit/, GFAP/Synaptic Systems, Göttingen, Germany, produced in mouse/) overnight at 4°C. The following day the cells were incubated with the appropriate secondary antibodies (120 min. RT). Finally, the cell nuclei were stained with DAPI. Immunofluorescent images were acquired by an Olympus FV3000 confocal microscope with a 60× oil-immersion lens (NA: 1.4). Single 1-μm-thick optical sections were scanned from the cell cultures, the confocal settings (laser power, confocal aperture and gain) were identical for all methods. The scanned images were processed by Adobe Photoshop CS5 software.

### Fluorescent Double Immunostaining of Spinal Cord Sections

Non-treated C57BL/6 mice (*n* = 3) and CFA-treated mice on post-injection day 1 (*n* = 3) and on post-injection day 4 (*n* = 3) were deeply anesthetized with sodium pentobarbital intraperitoneally (50 mg / kg.) and transcardially perfused with Tyrode’solution (oxygenated with a mixture of 95% O2, 5% CO2), followed by a fixative (4% paraformaldehyde dissolved in 0.1 M phosphate buffer/PB, pH 7.4/). After transcardial fixation the lumbar segments of the spinal cord were removed and placed into the same fixative for an additional 4–5 h, and immersed in 10 and 20% sucrose dissolved in 0.1 M PB until they sank. In order to aid reagent penetration the spinal cord was freeze-thawed in liquid nitrogen. After cryoprotection tissue pieces were embedded into agarose, and the L4–L5 segments of the spinal cords were sectioned at 50 μm on a vibratome, followed by extensive washing in 0.1 M PB.

In order to study the co-localization of GFAP with the cytokine markers double immunolabelings were performed. Before staining with the primary antibodies tissue sections were kept in 10% normal donkey serum (Vector Labs) for 50 min. Free-floating sections were then incubated with a mixture of antibodies that contained (a) GFAP (diluted 1: 2000, Synaptic Systems, produced in mouse) and one of the following antibodies: (b) anti-IL-6 (1:500, Peprotech, produced in rabbit), (c) anti-GM-CSF (1:500, Peprotech, produced in rabbit), (d) anti-CCL5 (1:500, Peprotech, produced in rabbit). The sections were kept in the primary antibody mixtures at 4°C for 2 days and then they were placed into the solution of secondary antibodies for 2 h (goat anti-mouse IgG conjugated with Alexa Fluor 488/diluted 1:2000, Invitrogen/and goat-anti-rabbit IgG conjugated with Alexa Fluor 555/diluted 1:2000, Invitrogen/). Finally, sections were mounted on glass slides and covered with Vectashield mounting medium (Vector Labs).

Single 1-μm-thick optical sections were scanned with an Olympus FV3000 confocal microscope. Scannings were performed with a 10× objective lens (NA: 0.4). All the setting (laser power, confocal aperture) were the same for all scans. Images obtained from three non-treated, and six CFA-injected animals (3 on post-injection day 1 and 4, respectively) were processed with Adobe Photoshop CS5 software. By filtering the background staining, basal threshold values were set for both GFAP and the other markers.

### Quantitative Analysis of the Spinal Cord Sections

The confocal fluorescent z-stack sections of spinal dorsal horn specimen were captured with 60× oil-immersion lens (NA: 1.4) of an Olympus FV3000 confocal laser microscope. All confocal settings were adjusted with the same parameters (confocal aperture, laser aperture- and intensity). The obtained image stacks were further analyzed with IMARIS software (Bitplane), which evaluated the co-localization data between the immunoreactive spots of IL-6, GM-CSF, CCL5 cytokines and the GFAP labeled astrocyte profiles rendered by the algorithm based on the staining in a standard way. The co-localization was validated if the detected spots and astrocyte surfaces were within the distance of 0.3 μm from each other. The analysis was carried out on five randomly selected images at each marker taken from control and treated superficial spinal dorsal horn sections of day 1 and 4 following CFA injection, respectively. The co-localization numbers for each cytokine and time point were averaged and standard error of means were calculated. Statistical differences between groups was analyzed by the Kruskal-Wallis statistical probe followed by Mann-Whitney pairwise comparison.

### Cell Treatment and Western Blotting

10–12-day-old astrocyte cultures were stimulated with different concentrations of IL-1β (ranging between 0.1 and 100 pg/ml) for variable durations (between 2 and 24 h). For NF-κB inhibition the cultures were treated with BAY-11-7082 for 4 h together with 10 ng/ml IL-1β in PBS. The SN50 peptide was used as a pretreatment for 1 h which was followed by 4, 8 or 16 h of IL-1β application. After the treatments the culture supernatants were removed and the proteins of 200 μl of supernatant was precipitated with two-times amount of ice cold acetone. The precipitated proteins were separated by centrifugation (10 min. 3000 rpm 4°C). Cells attaching the culture plates were lysed and cytosol and nuclear fractions were separated as it has been already described. Supernatants and cytosol/nuclear fractions were kept in aliquots at −70°C until they are used for western blotting.

Briefly, the supernatants or cellular fractions were mixed with reducing sample buffer (1:1 ratio) and run on 12% SDS-polyacrylamide gels. The separated proteins were electroblotted onto PVDF membranes. After blocking the unspecific labeling with 10% BSA solution, the membranes were treated with anti-primary antibody (anti-IL-6, anti-GM-CSF, anti-CCL5/Peprotech, produced in rabbit/; anti-p50, anti-ikB/Santa Cruz, produced in mouse/; anti-p65/Invitrogen, produced in mouse/GFAP/Synaptic Systems, produced in mouse/) followed by incubation with the corresponding goat-anti-rabbit-HRP or goat-anti-mouse-HRP (DAKO, Glostrup, Denmark) secondary antibody. Finally, the labeled bands were visualized by 3, 3′-diaminobenzidine.

### Enzyme Linked Immunosorbent Assay (ELISA)

ELISA was performed as already described (Holló et al., 2000). Briefly, (after titration in increasing dilutions) the astrocyte culture supernatants were diluted in 1:1 ratio by ELISA coating buffer (15 mM Na_2_CO_3_, 35 mM NaHCO_3_, pH 9.6) and used for coating of 96 well polystyrol ELISA plates (Maxisorp, NUNC Intermed, Copenhagen, Denmark). Free binding capacity of the wells was blocked by 1% gelatin (Reanal, Budapest, Hungary) in PBS. After washing with PBS anti-IL-6, anti-GM-CSF or anti-CCL-5 primary antibodies were added (1:1000, 2 h, 37°C), followed by goat-anti-rabbit-HRP secondary antibody (1:500, DAKO). Color reaction was developed by o-phenylenediamine and the optical density was measured at λ = 492 nm by ELISA plate reader (Titertek, Uniscan). Graphs represent results of three independent experiments.

### Antibody Controls

The cytokine antibody specificity was tested on control spinal cord sections by antibody depletion as it has been described earlier (Holló et al., 2017). Briefly, the diluted anti-IL-6, anti-GM-CSF, anti-CCL5 antibodies were mixed with recombinant murine IL-6, GM-CSF and CCL5 peptides (PeproTech), respectively. The mixtures and also diluted primary antibodies alone, were incubated at 4°C for 18 h and then centrifuged (4°C, 30,000*g*, 30 min). Thereafter the spinal cord sections were treated with one of the cytokine antibodies or with one of the pre-incubated mixtures for 48 h at 4°C, then placed into biotinylated goat anti-rabbit IgG dissolved in TPBS (diluted 1:200, Vector Labs, Burlingame, United States) for 4 h at room temperature. The color reactions were developed by 3,3′- diaminobenzidine. Photomicrographs were taken by Olympus CX-31 epifluorescent microscope with fixed settings. The pre-adsorption of cytokine antibodies with the appropriate cytokine proteins abolished the specific immunostaining (Supplementary Material).

## Results

### Mechanical Pain Sensitivity of C57/BL6 Mice During the Course of CFA-Induced Inflammatory Pain

**FIGURE 1:**
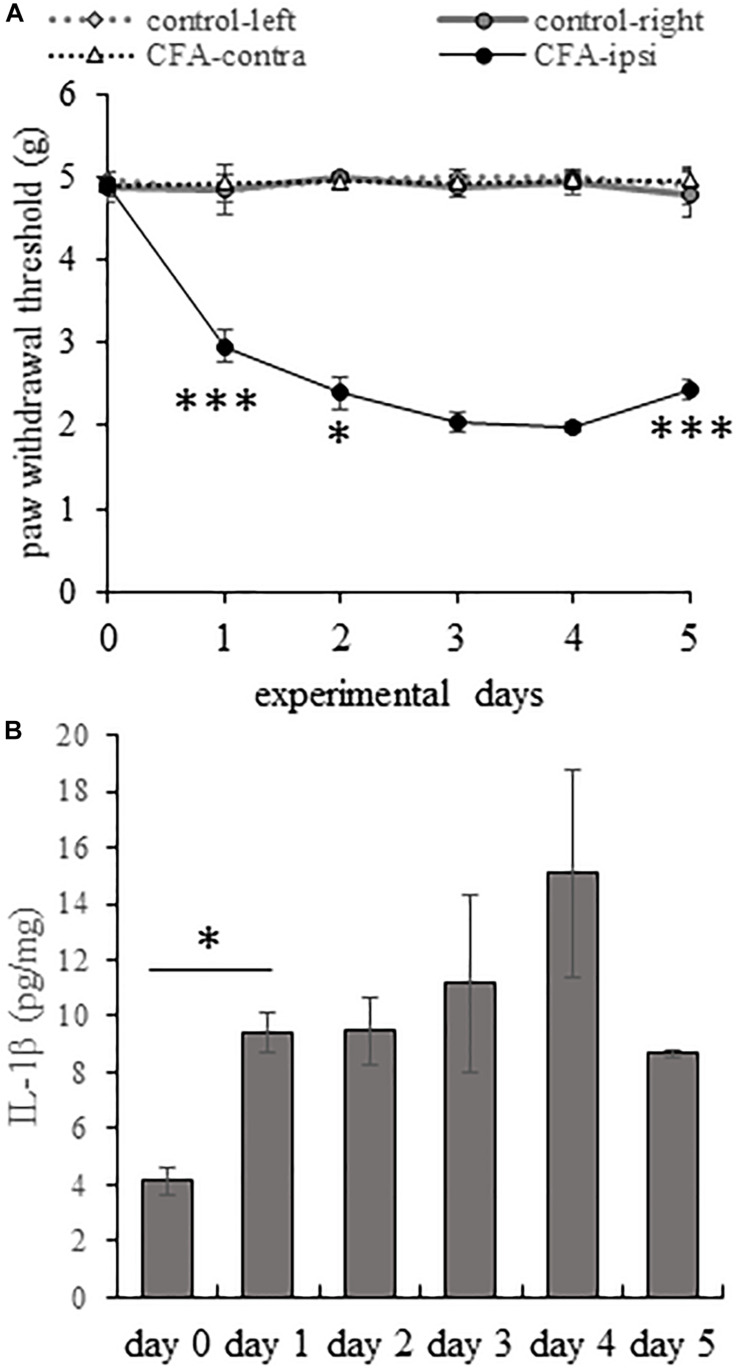
During the course of CFA-induced inflammatory pain IL-1β production correlates with enhanced mechanical pain sensitivity. **(A)** Line chart shows the mean mechanical withdrawal threshold (MWT) values on both hind limbs of control animals and animals receiving CFA injection into the right (ipsilateral) hind paw. Note that MWT values appeared to be remarkably stable throughout the entire length of the experimental period in control animals and in the untreated left (contralateral) hind paw of animals receiving CFA injection. However, CFA injection resulted in a significant drop in MWT values in the ipsilateral hind paw of animals on experimental days 1–4. Mechanical pain sensitivity peaked on post-injection day 3 and 4. Data are presented as mean ± SEM. (One way ANOVA, ^∗∗∗^*p* < 0.001; ^∗^*p* < 0.05, asterisk show results of comparison of two consecutive days). **(B)** Bar chart shows quantitative ELISA measurement of IL-1β levels in tissue extracts obtained from the L4–L5 lumbar segments of the spinal dorsal horn of the control (day 0) and CFA-injected animals at post-injection days 1–5. Data are shown as mean ± SEM. Note that the cytokine level reached significant elevation on post-injection days 1–5 (One way ANOVA, ^∗^*p* < 0.05).

The mechanical withdrawal threshold (MWT) was very similar in all animals before CFA injection (4.91 ± 0.039 g). On the treated, right (ipsi-lateral) hind paw, the CFA-treatment induced a highly significant increase in pain sensitivity on the first experimental day, MWT dropped to 2.96 ± 0.052 g (*p* = 0.000101). The reduction of the MWT values continued until day 3 and 4 when MWT values decreased to 2.03 ± 0.117 and 1.99 ± 0.065 g, respectively. There was no statistically significant difference between MWT values on day 3 and day 4. We followed the measurement for an additional day and we found that on day 5 the nociceptive sensitivity significantly attenuated (*p* = 0.000381), MWT values reached 2.43 ± 0.118 g. However, this significant change was not observed on the left (contra-lateral) side and the MWT levels were stable through the course of the experiment (Figure 1A).

According to other authors (Hylden et al., 1989; Pitzer et al., 2016) the drop in MWT values peaks on the third day after CFA administration, which is in accord with our observations.

### Peripheral Inflammation Evoked by CFA Injection Induced Elevation of Spinal IL-1β Expression

Although the expression of IL-1β has already been demonstrated in the spinal cord (Raghavendra et al., 2004; Liu et al., 2008), there has been no data in the literature to follow the time dependent changes in the expression of the cytokine in inflammatory pain. Thus, we intended to explore how the expression of IL-1β changes in CFA-induced inflammatory pain at the protein level (Figure 1B) in the spinal dorsal horn tissue extract of the L4–L5 spinal segments, which is known to receive primary afferent inputs from the plantar surface of the hind paw (Molander and Grant, 1985).

Measuring the quantity of IL-1β protein with the quantitative ELISA method we found that CFA-evoked plantar inflammation induced a significant elevation in the expression of IL-1β. The basal level of the cytokine was 4.15 ± 0.43 pg/mg in the spinal dorsal horn tissue extract of the L4–L5 spinal segments which significantly (*p* = 0.049) increased to 9.43 ± 0.73 pg/mg on experimental day 1, correlating the significant drop of MWT measured on the ipsi-lateral hindpaw of the CFA injected animals on the same day. Highest cytokine level was measured on day 4 (15.08 ± 3.66 pg/mg) corresponding the highest mechanical pain sensitivity. We followed the cytokine level for an additional day and observed that on day 5 of the experiment the IL-1β concentration dropped to 8.69 ± 0.12 pg/mg. Our earlier experiments showed that the mechanical pain sensitivity gradually attenuates after day 5 and finally returns to the basal level on post-injection day 11 (Holló et al., 2017).

We found in the literature only one paper where the IL-1β content of spinal cord is given to the weight of the tissue. Wang et al. (1997) found lower level of the cytokine in the control rat spinal cord. This difference can be due several reasons e.g., species differences between rats and mice, or due to the different sample collection as they used the entire spinal cord for the measurement while we extracted only the dorsal horn tissue.

Significant changes in the nociceptive behavior and robust elevation of the IL-1β production in the spinal cord suggest that the first 24 h is a very critical period during the course of the CFA-evoked pain. Thus, in the further experiments we detected the activation and cytokine/chemokine secretion of spinal astrocyte cultures in this period.

### Cultured Spinal Astrocytes Express the Ligand Binding Unit of the IL-1 Receptor (IL-1R1) and They Are Activated by IL-1β in a Concentration-Dependent Way

Astrocytic activation is increasingly accepted as a factor which contributes to chronic pain states through its contribution to central sensitization. Thus we wanted to investigate astrocytic activation using IL-1β as a stimulating agent which is upregulated in the spinal cord during inflammatory pain and its upregulation correlates with the nociceptive sensitivity during the course of CFA-evoked pain.

**FIGURE 2:**
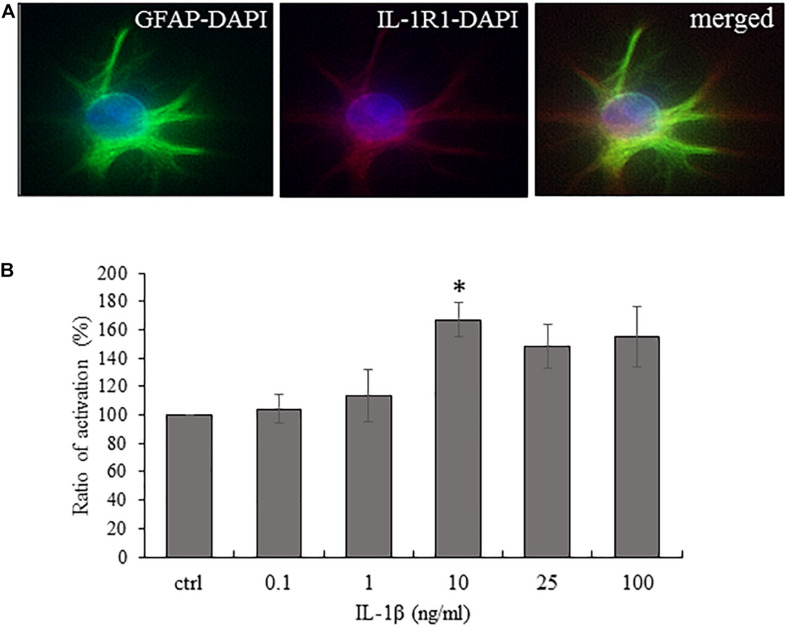
Cultured spinal astrocytes express IL-1R1 and their activity is significantly increased upon IL-1β stimulation. **(A)** Cultured spinal astrocytes express the ligand binding unit or IL-1 receptor (IL-1R1). Micrographs of fluorescent images illustrating co-localization between GFAP astrocytic marker (a,c; green) and IL-1R1 (b,c; red). Panel a–c represent control cultures. Mixed colors (yellow) on the superimposed image (c) indicate double labeled structures. On all images DAPI was used to label cell nuclei (blue). **(B)** Dose-dependent (1–100 ng/ml) enhancement of astrocytic activity by IL-1β was determined by MTT assay after 24 h of treatment. Quantification of MTT activity is presented as fold change over the control cells in percentage. Data are shown as mean ± SEM of three independent experiments in duplicate assay (ANOVA Repeated Measures, followed by Tukey’s pairwise comparison ^∗^*p* < 0.05 versus control group).

Although we and others (Wang et al., 2006; Holló et al., 2017) already showed that the spinal astrocytes express IL-1R1, we confirmed its expression on the cultured astrocytes (Figure 2A).

As a next step, cell activity (MTT) assay was performed to identify the lowest IL-1β concentration which is suitable to increase astrocytic activity significantly. We used serial dilutions (1–100 ng/ml) of the recombinant IL-1β protein and found that 24 h stimulation with 10 ng/ml of the protein increased significantly the cellular activity to 167 ± 12.23% (*p* = 0.02815) of the control level (Figure 2B). Thus, we used this concentration of the cytokine in the further stimulation experiments. As a negative control, we also tested the IL-1β responsiveness of spinal astrocyte cultures isolated from IL-1R1 knock-out mice and we found no significant upregulation of mitochondrial activity (data not shown).

### Spinal Astrocytic Secretome Profile Reveals IL-1β-Induced Overexpression of 3 Cytokines and Chemokines Out of the 24 Produced by Non-treated Astrocyte Cultures

**FIGURE 3:**
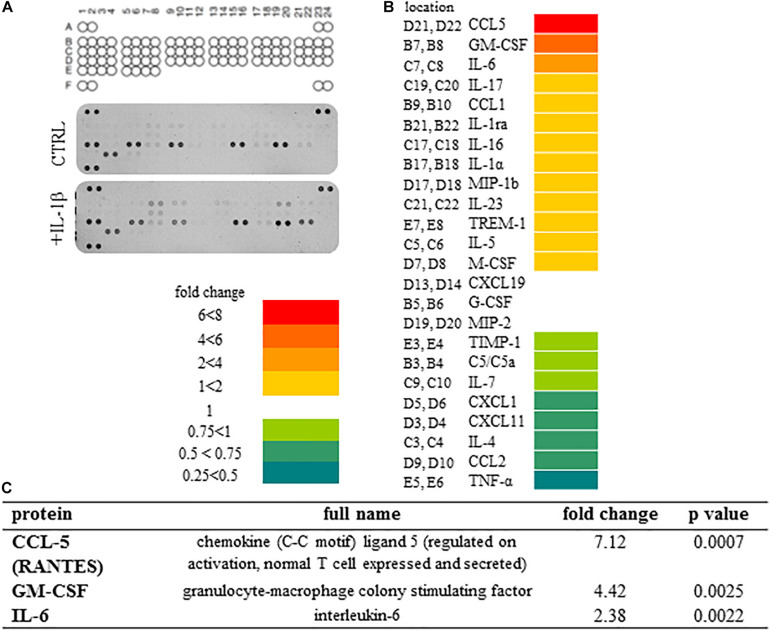
Secretome profile of IL-1β treated spinal astrocytes reveals the expression of 24 cytokines and chemokines. **(A)** Coordinates of cytokines on array membranes and developed membranes. The Proteome Profiler array used in the experiments allowed the detection of 40 cytokines, chemokines and other soluble factors. Upper panel shows array coordinates. Middle panel shows expression of cytokines/chemokines in the supernatant of unstimulated spinal astrocytes, and lower panel shows expression of cytokines/chemokines by IL-1β stimulated astrocytes. **(B)** Heat map analysis of spinal astrocytic cytokine profile after 24 h of IL-1β stimulus. Left column shows array coordinates of cytokines/chemokines in the middle column and right column shows the fold change of their amount. **(C)** List of cytokines/chemokines which are significantly overexpressed in the supernatant of spinal astrocyte cultures upon IL-1β stimulation.

We detected the secretion of 24 cytokines/chemokines in the supernatant of spinal astrocyte cultures out of the 40 molecules included into the assay (Figure 3A). When comparing the cytokine levels in culture supernatants obtained from control and IL-1β-treated spinal astrocytes, we observed the production of the same cytokines/chemokines, we have not detected any newly synthetized molecules. We, however, found significant upregulation of three molecules: CCL5 (RANTES), GM-CSF, IL-6. Highest, approximately seven fold change was measured for the production of CCL5 (RANTES), which was also highly significant (*p* = 0.0002) if compared with the control level of the chemokine. GM-CSF levels exceeded approximately four fold (*p* = 0.0025) and IL-6 levels approximately 2 fold (*p* = 0.0022) the control levels of the cytokines, respectively (Figure 3B).

**FIGURE 4:**
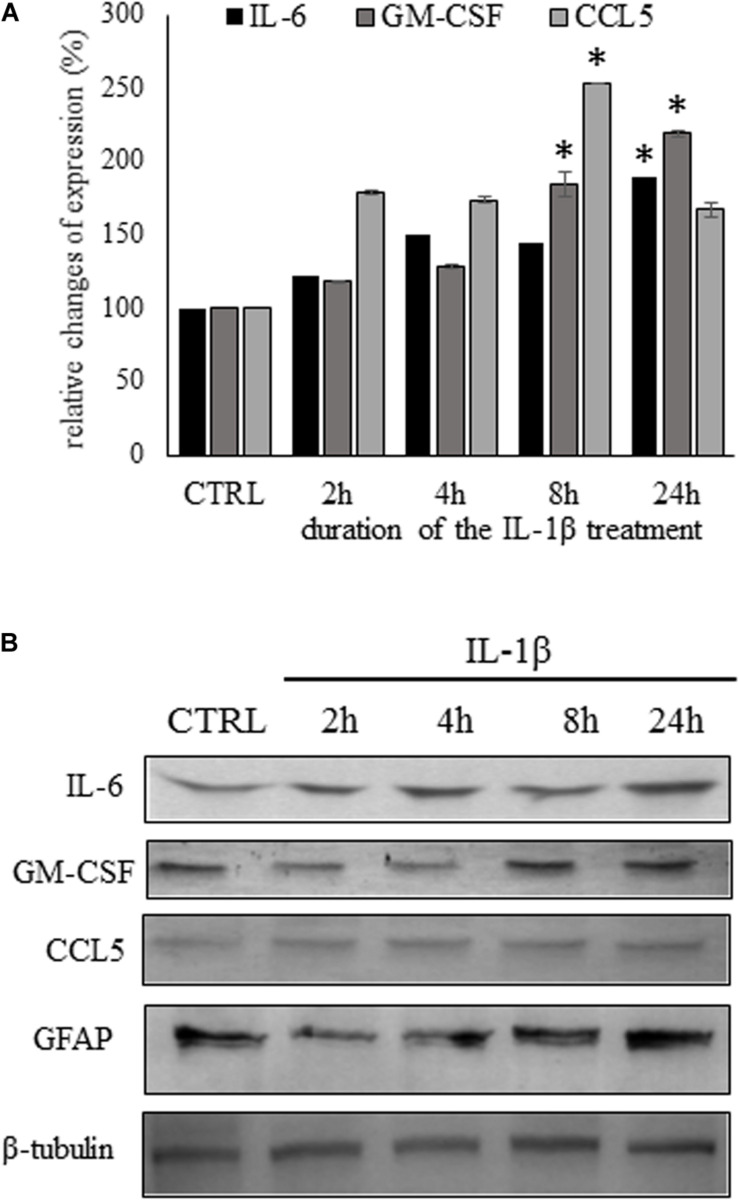
Time course experiments show different kinetics of cytokine secretion due to IL-1β stimulation. **(A)** Secretion of pro inflammatory cytokines was significantly increased in the supernatant of IL-1β stimulated astrocytes by ELISA experiments. Data are shown as mean ± SEM of three independent experiments in duplicate assay. ANOVA Repeated Measures, followed by Tukey’s pairwise comparison, ^∗^*p* < 0.05 versus control group. **(B)** Representative immunoblots of IL-6, GM-CSF, and CCL5 expression in the supernatans of astrocyte cultures. In the cytosol of astrocyte cultures GFAP expression also shows gradual time-dependent enhancement.

We followed the time-dependent changes of the three chemokine/cytokine levels during the critical 24 h as we observed in the spinal cord the significant enhancement of IL-1β secretion during this period. The time-course experiments showed slightly different secretion pattern of the three molecules. Early activation of CCL5 secretion, was observed even after 2 h of IL-1β treatment it considerably elevated level (179.0 ± 2.59%) and it reached its peak after 8 h of stimulation (253.4 ± 0.5.6%, *p* = 0.006272). While the IL-6 and GM-CSF activation was slower, increased gradually until the end-point of the experiment. At this time the two cytokine levels were significantly elevated if compared with the control level (IL-6: 189.5 ± 1.74%, *p* = 0.002369; GM-CSF: 219.3 ± 2.35%, *p* = 0.000209), in accordance with the Protein Profiler data (Figure 4A).

We also carried out western blot analysis from the supernatants of the control and IL-1β-stimulated cultures for the detection of the three significantly upregulated cytokines. We observed immunoreactive bands at the expected molecular weight of each cytokine/chemokine. The changes in cytokine levels, observed on the western blots, were comparable of the data received by the ELISA experiments. In the cell lysates of the cultures IL-1β-induced gradual increase of GFAP expression, similarly as it has been described previously by other authors (Liddelow et al., 2017; Yang et al., 2019; Figure 4B).

**FIGURE 5:**
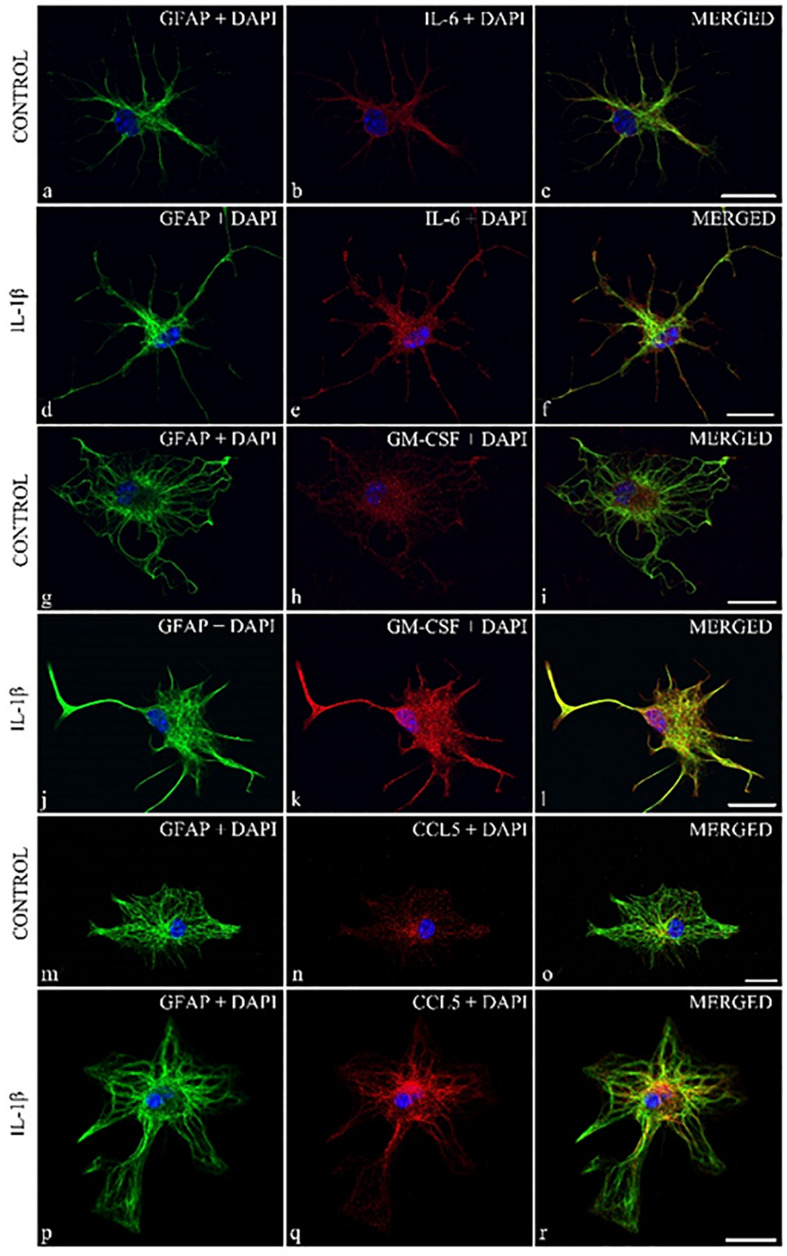
Localization of cytokines in cytoplasmic compartment of GFAP + spinal astrocyte cultures: enhanced cytokine expression was observed in IL-1β stimulated spinal astrocytes. Micrographs of single 1 μm thick laser scanning confocal optical sections illustrating co-localization between GFAP astrocytic marker (green) and IL-6, GM-CSF, CCL5 cytokines (red). Panels **(a–c**,**g,h**,**l–n)** represent control, while **(d–f,i–k,o–q)** show cultures which received 24 h of IL-1β treatment. Mixed colors (yellow) on the superimposed images **(c,f,h,k,n,r)** indicate double labeled structures. On all images DAPI was used to label cell nuclei (blue). Scale bar 10 μm.

To further demonstrate the astrocytic expression of the cytokines, we also performed double immunostainings on control astrocytes and on cultures which received 24 h of IL-β-treatment. The 1 μm thick optical images showed co-localization of the three cytokines/chemokines with the GFAP marker in the control cultures and enhanced expression of the cytokines and GFAP in the IL-1β-stimulated astrocytes (Figure 5).

### Spinal Expression of IL-6, GM-CSF and CCL5 Is Increased During the Course of CFA-Evoked Inflammatory Pain

To determine if the three selected cytokines were also overexpressed in the spinal dorsal horn upon peripheral CFA-injection, spinal cord sections were prepared from the L4–L5 segments which receive primary afferent fibers from the hindpaws. In control sections low levels of cytokines were detected which was mostly restricted to the superficial laminae (corresponding to Rexed laminae I and II, Figures 6.1–6.3, 6.10–6.12, 6.19–6.21). To follow the time-dependent changes of the selected cytokines during the course of the pain model we obtained confocal images from the initial phase of the model (post-injection day 1) and at the time of highest mechanical sensitivity (post-injection day 4). Interestingly, in case of IL-6 and GM-CSF we could observe considerable elevation of cytokine expression on both sides of the spinal cord, on the first post-injection day. The intensity of the immunoreactivity was increased further on post-injection day 4 and was more pronounced on the ipsi-lateral (right) side (Figures 6.4–6.9, 6.13–6.18). The CCL5 chemokine showed different time course: on the first post-injection day just a moderate enhancement of the CCL5 + signal was visible (Figures 6.22–6.24), and it was only on post-injection day 4 (Figures 6.25–6.27) when the pronounced elevation of the chemokine was detected in the superficial layers of the ipsi-lateral (right side) spinal dorsal horn.

**FIGURE 6:**
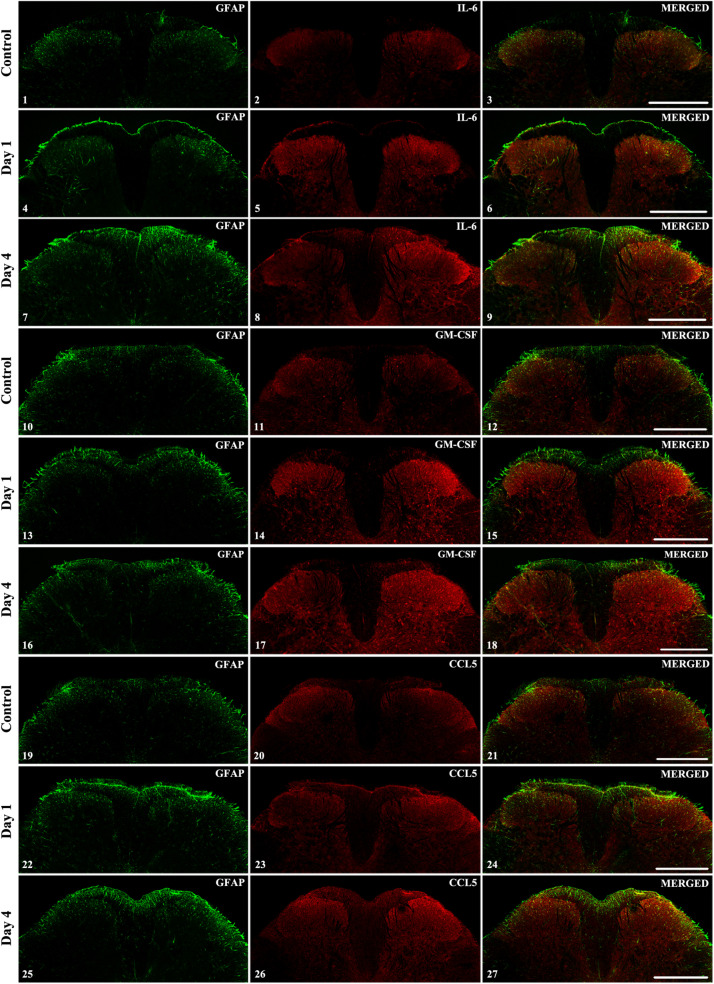
The spinal expression of IL-6, GM-CSF, and CCL5 cytokines during the course of CFA-evoked pain. Representative 1 μm thick confocal images illustrating the co-localization between immunolabeling for IL-6 (red; 6.2, 6.5, 6.8), GM-CSF (red; 6.11, 6.14, 6.17) CCL5 (red; 6.20, 6.23, 6.26) and the immunoreactivity of astrocytes (GFAP, green; first column of the figures) in the superficial spinal dorsal horn. Mixed colors (yellow) on the superimposed images (last column of the figures) indicate double-labeled structures. For each cytokine the first row of the images are taken from control samples, whereas the second row of images represents the first day after CFA injection and the third row shows the spinal expression of the cytokines on post-injection day 4. Increased labeling of the cytokines was apparent on the ipsi-lateral (right side) of the spinal cords on post-injection day 4 (panels 6.8, 6.17, and 6.26). In each case scale bars: 500 μm.

### Quantitative Analysis of Spinal IL-6, GM-CSF and CCL5 Expression and Co-localization With GFAP Marker During the Course of CFA-Induced Pain Model

**FIGURE 7:**
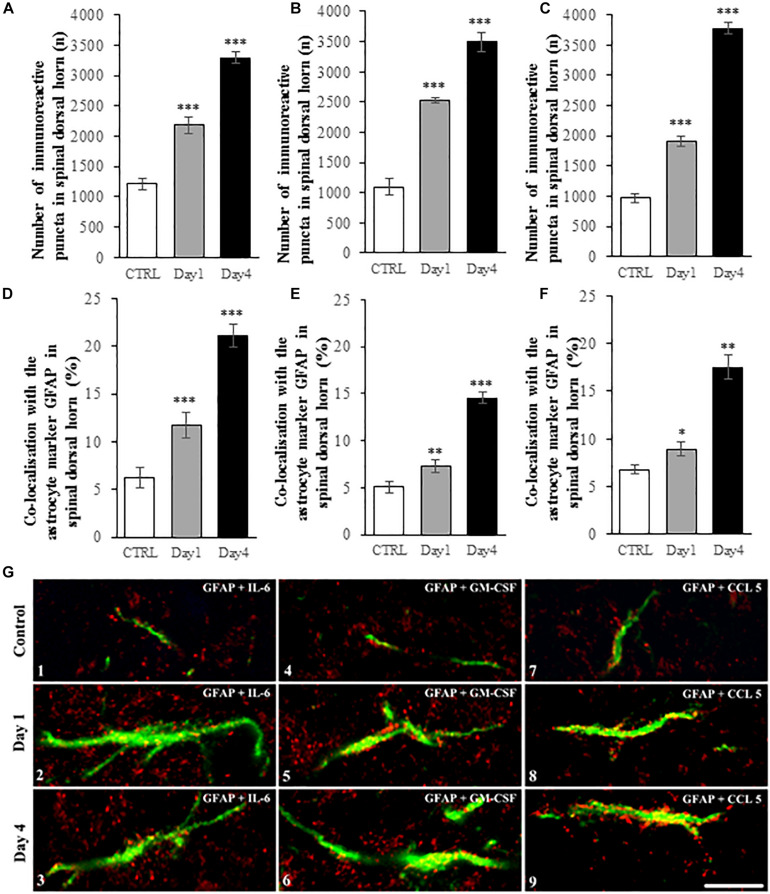
IMARIS analysis of spinal cord sections show enhanced expression IL-6, GM-CSF, and CCL5 and increased co-localization of the cytokines with GFAP astrocyte marker during the course of CFA-evoked inflammatory pain. **(A–C)** Histograms show significant elevation of IL-6 **(A)**, GM-CSF **(B)** and CCL5 **(C)** immunoreactive puncta in spinal dorsal horn on post-injection days 1 and 4. Data are shown as mean ± SEM of five randomly selected spinal cord specimen (ANOVA, followed by Tukey’s pairwise comparison ***p < 0.001 versus control group). **(D–F)** Bar charts represent the ratio of co-localization between the IL-6 **(D)**, GM-CSF **(E)** and CCL5 **(F)** cytokine and GFAP in control and treated (on post-injection day 1 and 4) spinal cord specimen. Data are shown as mean ± SEM of five randomly selected spinal cord specimen. (Kruskal-Wallis statistical probe, followed by Mann-Whitney pairwise comparison ^∗^*p* < 0.05, ^∗∗^*p* < 0.01, ^∗∗∗^*p* < 0.001 versus control group). **(G)** Representative high magnification superimposed confocal images of GFAP + astrocyte profiles (green) and IL-6 (red, G.1-3), GM-CSF (red, G.4-6) and CCL5 (red, G.7-9) immunoreactive puncta. First row of images (G.1, G.4, G.7) represents control specimen. Second (G.2, G.5, G.8) and third (G.3, G.6, G.9) rows show specimen on post-injection day 1 and 4, respectively. Mixed color (yellow) indicates co-localization of the markers. Scale bar: 10 μm.

IMARIS software analysis shows that peripheral CFA injection significantly enhanced the number of IL-6, GM-CSF and CCL5 immunoreactive spots on experimental day 1 and 4 in spinal dorsal horn. Changes in the absolute numbers of immunoreactive puncta (Figures 7A–C) was in all cases highly significant (*p* < 0.001). Similarly, the number of co-localized cytokine spots on astrocytes was found to be significantly higher in the CFA-treated animals compared to control animals (Figures 7D–F). The co-localization between GFAP and IL-6, GM-CSF and CCL5 was quite similar in control conditions (6.25 ± 1.03%, 5.04 ± 0.64%, and 6.79 ± 0.48%, respectively). However, after the CFA-treatment the ratio of co-localization was highest between IL-6 cytokine and GFAP marker both on day 1 (11.65 ± 1.33%, *p* = 0.00085) and on day 4 (21.10 ± 1.21%, *p* = 0.00019). The enhancement in co-localization values for the other two cytokines were more moderate, but still significant. On experimental day one the co-localization with GFAP profiles increased to 7.33 ± 0.69% (*p* = 0.002) in case of GM-CSF and to 8.93 ± 0.77% (*p* = 0.012) in case of CCL5. The co-localization values for the latter two cytokines significantly elevated further on experimental day 4 (GM-CSF: 14.57 ± 0.064%, *p* = 0.00019; CCL5: 17.53 ± 1.26%, *p* = 0.00012).

We also demonstrated on high magnification confocal images the co-localization between the three studied cytokines and the GFAP+ astrocytic profiles (Figures 7G 1–9).

### IL-1β Activates the NF-κB Pathway in Spinal Astrocytes

IL-1β signaling is associated with the NF-κB and MAPK pathways (Medzhitov, 2001). As astrocyte-specific activation of the NF-κB pathway upon IL-1β stimulation has already been reported by Srinivasan et al. (2004) in the hippocampus, we intended to explore the role of the pathway in the spinal astrocyte cultures.

**FIGURE 8:**
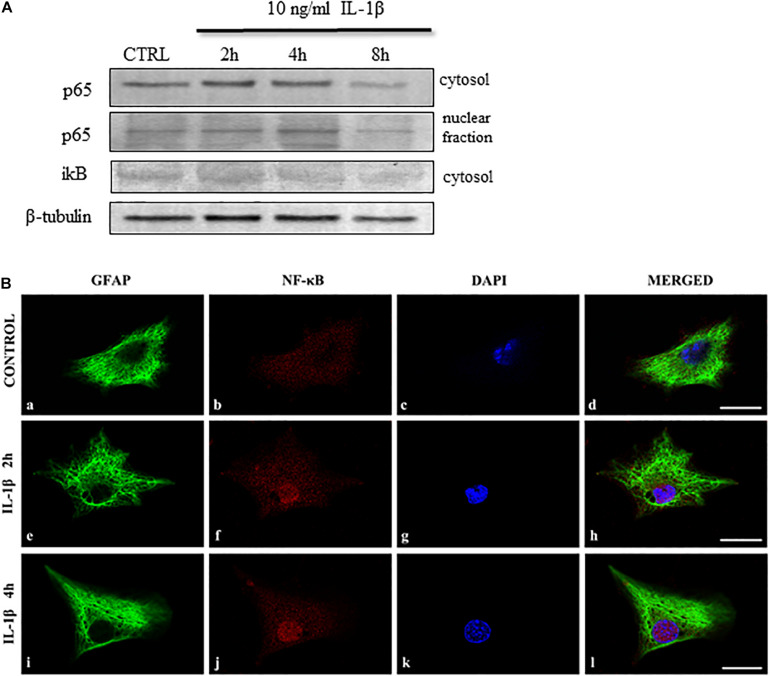
IL-1β activates the NF-κB signaling pathway in spinal astrocyte cultures. **(A)** Representative immunoblots show time-dependent changes of the NF-κB signaling pathway upon IL-1β stimulation of spinal astrocyte cultures. NF-κB p65 expression increase after 2 h of stimulation in the cytosol and the p65 protein appears in the nuclear fraction after 2 h of treatment. The inhibitory iκB unit is downregulated in the cytosol of the cultures upon 4 h of stimulation. **(B)** Micrographs of single 1 μm thick laser scanning confocal optical sections illustrating cytoplasmic expression [GFAP astrocytic marker (green)] of p65 protein in control cultures (panels **a–d**). The nuclear translocation of the p65 protein was observed after 2 (panels **e–h**) and 4 h (panels **i–l**) of IL-1β treatment. On all images DAPI was used to label cell nuclei (blue). Mixed color (purple) on the superimposed images (**d,h,l**) indicate double labeled structures. Scale bars: 10 μm.

To reveal the effect of IL-1β on the NF-κB signaling we studied several members of the pathway in the astrocyte cell lysates (Oeckinghaus et al., 2011). We detected the cytosolic and nuclear levels of the p65 (RelA) and the inhibitory iκB. When following the time course of these IL-1β-induced NF-κB activation we found the elevation of the NF-κB p65 protein in the cytosol of astrocytes within 2 h. The translocation of p65 protein to the nucleus occurred after 4 h of stimulation and it was paralleled by the decrease of the inhibitory iκB unit in the cytosol (Figure 8A). The nuclear translocation of the p65 protein was also demonstrated by immunohistological staining of astrocyte cultures, which showed the presence of the protein after 2 h of IL-1β stimulation (Figures 8B e–h) and increased further after 2 additional hours of treatment (Figure 8Bi-l). These data demonstrate the IL-1β-induced activation of the NF-κB pathway in the spinal astrocytes.

### IL-1β-Induced IL-6 Secretion Is Significantly Suppressed by the NF-kB Inhibitor BAY 11-7082

**FIGURE 9:**
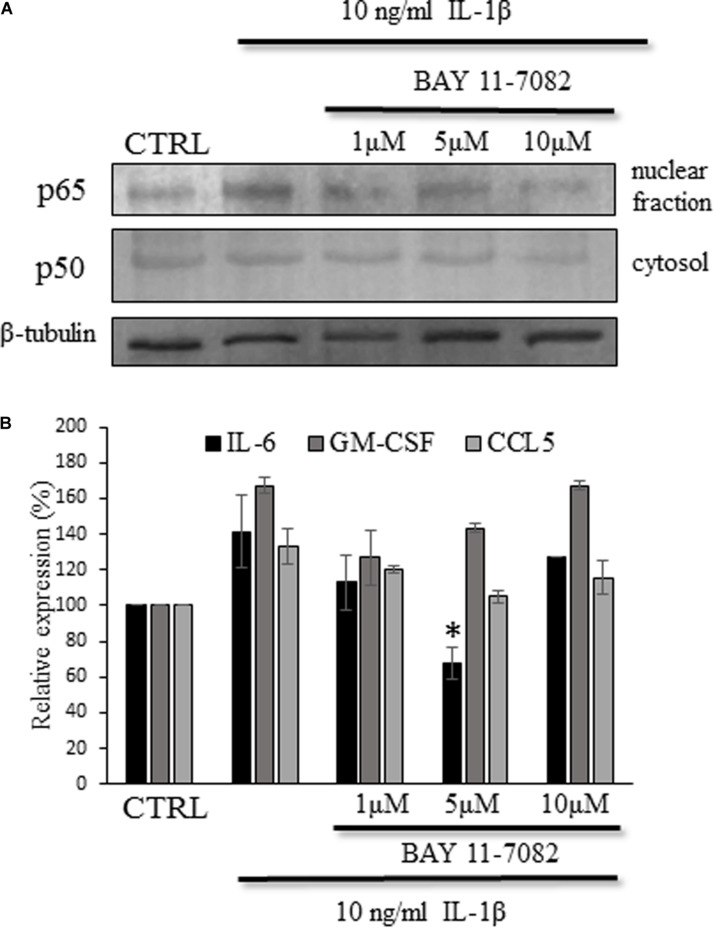
IL-6 expression was attenuated by the BAY 11-1082 inhibitor of the NF-κB signaling pathway. **(A)** Representative immunoblot show decreased p65 expression in the nuclear fraction upon BAY 11-1082 treatment. **(B)** Histogram shows cytokine levels determined by ELISA method. The IL-6 production of the astrocyte cultures is significantly attenuated. Data are shown as mean ± SEM of three independent experiments in duplicate. One-way ANOVA, followed by Student-Newman-Keuls pairwise comparison, ^∗^*p* < 0.05.

To confirm the role to NF-κB activation in the IL-1β induced cytokine/chemokine secretion we utilized the NF-κB inhibitor BAY 11-7082, which is an irreversible inhibitor of iκB-α phosphorylation resulting in the inactivation of NF-κB pathway (Pierce et al., 1997; Phulwani et al., 2008). As in the previous experiments we detected the nuclear translocation of the p50 protein after this time period, the cultures were treated for 4 h with IL-1β and different concentrations (ranging between 1 and 10 μM) of the NF-κB inhibitor. To validate the experimental system we confirmed that the inhibitor blocks the activation of NF-κB and we observed decrease in the cytosolic p50 and nuclear p65 proteins upon treatment with the inhibitor (Figure 9A). When measuring the cytokine levels in the supernatant of the cultures we observed decreased concentrations in the BAY 11-7082-treated culture supernatants, but it reached significant level only in case of IL-6 production (67.3 ± 8.6%, *p* = 0.009) (Figure 9B).

### IL-1β-Induced IL-6, GM-CSF, CCL5 Secretion Is Significantly Suppressed by the NF-kB Inhibitor SN50 at Different Time Points

**FIGURE 10:**
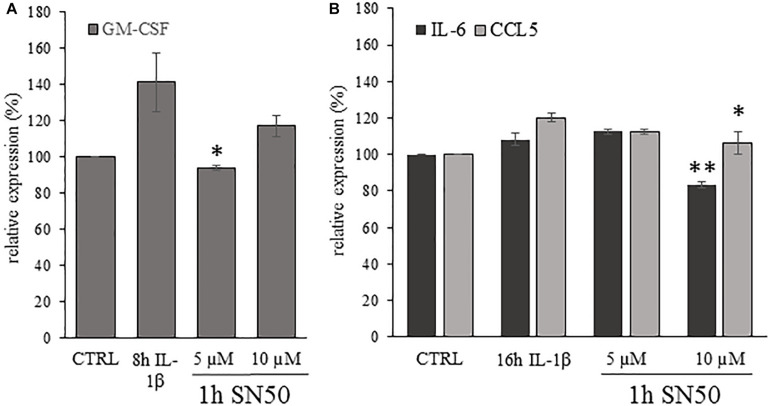
Astrocytic cytokine expression was significantly reduced by SN50, inhibitor of NF-κB nuclear translocation. **(A)** Histogram shows relative GM-CSF levels determined by ELISA method after 1 h of pre-treatment by SN50 and 8 h of IL-1β stimulation. The GM-CSF secretion of the astrocyte cultures is significantly down regulated by 5 μM SN50 if compared with the IL-1β-treated cultures. **(B)** Histogram shows relative IL-6 and CCL5 levels in the supernatant of the astrocyte cultures measured by ELISA method after 1 h of SN50 pre-treatment and 16 h of IL-1β stimulation. Both cytokine levels show significant reduction due to 10 μM SN50 treatment if compared with the IL-1β-treated cultures. Data are shown as mean ± SEM of two independent experiments in duplicate. One-way ANOVA, followed by Student-Newman-Keuls pairwise comparison, ^∗^*p* < 0.05; ^∗∗^*p* < 0.01.

As a second approach to influence NF-kB signaling we used the SN50 peptide, which is a cell permeable inhibitor of NF-kB nuclear translocation (Lin et al., 1995). Its specificity for the NF-kB pathway is confirmed in lower concentration (Boothby, 2001)., thus we used the SN50 peptide for 1 h pre-treatment of the astrocyte cultures in 5 and 10 μM concentrations, which was followed by 4, 8 or 16 h of IL-β stimulation. We could detect significant attenuation of the GM-CSF expression of the SN50 pre-treated cultures after 8 h of IL-1β treatment if compared to the IL-1β-treated cultures. The relative GM-CSF expression values of the IL-β-treated cultures was 141.5 ± 16.21% which dropped to 94.14% ± 1.22%; (*p* = 0.02) upon 5 μM SN50 pre-treatment (Figure 10A). For IL-6 and CCL5 the significant reduction of the cytokine levels was observed after 16 h of stimulation (Figure 10B). The IL-β-induced relative expression of IL-6 and CCL5 was 112.78 ± 3.77% and 120.24 ± 2.38%, respectively. These levels were significantly reduced due to 1 h pre-treatment with 10 μM SN50 (for IL-6 83.22 ± 3.57%; *p* = 0,002 and for CCL5 106.16 ± 6.03%; *p* = 0.043).

## Discussion

In summary, we found correlation of spinal dorsal horn IL-1β expression with the nociceptive behavior during the course of CFA-evoked inflammatory pain. We show that the stimulation of spinal astrocyte cultures by IL-1β results in significantly enhanced secretion of three inflammatory cytokines/chemokines: IL-6, GM-CSF and CCL5 (RANTES). The overexpression of the three selected cytokines was also confirmed in the spinal dorsal horn during the CFA-induced pain model. We studied the activation of the NF-kB signaling pathway which is associated with astrocyte-specific IL-1β signaling and we found its time-dependent activation during the course of IL-1β treatment. Finally, the inhibition of the NF-kB pathway resulted in the significant attenuation of cytokine production.

IL-1β exerts its action through its receptor which has a ligand binding unit (IL-1R1) and an accessory protein responsible for the signal transduction (IL-1RAcP) (Ren and Torres, 2009). The IL-1R1 was shown to be expressed by neurons and glial cells in the CNS. However, the ligand binding induce cell type specific responses, which is aided by the neuron-specific isoform of the IL-1RAcPb (Huang et al., 2011).

Gruber-Schoffnegger et al. (2013) suggested two possible ways how cytokines can act in the CNS: directly on neurons and indirectly by activating local glial cells to secrete further neuroactive substances which in turn modulate nerve cell functions. In this study our aim was to explore the latter way of cytokine actions by detecting the IL-1β-induced cytokine/chemokine production in spinal astrocytes.

Astrocytic activation can be induced by numerous agents including lipopolysaccharide (LPS) (Tarassishin et al., 2014), adenosine-triphosphate (ATP) (Adzic et al., 2017), glutamate (Aronica et al., 2005), cytokines etc. Upon different activation signals (or their combinations) astrocytes can turn into neurotoxic A1 or neuroprotective A2 phenotype. While in different pain states A1 astrocytes can release neurotoxins which can trigger the death of neurons and glial cells, A2 astrocytes induce cell survival and tissue regeneration (Li et al., 2019). Recently, however, it was suggested that astrocytic activation is possibly not an all-or-nothing phenomenon, but it is rather “gradated” (Matias et al., 2019). Liddelow et al. (2017) showed that in many cases the reactive astrocyte phenotype is not so polarized. Among others they showed that IL-1β stimulation induced upregulation of A1 and A2 marker genes. In another study neuroprotective aspects of IL-1β stimulated astrocytes was also revealed as genes encoding neurotrophic factors were upregulated in the cultures (Teh et al., 2017).

During CNS insults or injury both astrocytes and microglia are activated and their bidirectional communication can be important for their further fates and their possible role in CNS disorders. Microglia usually react faster to pathological stimuli and by their secreted molecules they can contribute to the consecutive activation of astrocytes (Jha et al., 2019). It was shown that IL-1β can be one of those microglia-derived factors which can lead to astrocytic activation. On the other hand, although astrocytic activation has a “lag phase,” it is more prolonged - in this way, they can have a role in the maintenance of enhanced pain states (Gao and Ji, 2010).

Interestingly, we found IL-1β-induced significant upregulation of pro-inflammatory cytokines/chemokines and we did not detect significant downregulation of any anti-inflammatory cytokines. All three identified molecules were shown previously to be expressed in different areas of the CNS.

IL-6 is classically described as a pro-inflammatory cytokine which induce T lymphocyte population expansion and B lymphocyte differentiation, but it is well described now that in a context-dependent way IL-6 may have anti-inflammatory functions as well (Hunter and Jones, 2015). Its ligand binding receptor (mIL-6R) is expressed on very limited cell types (e.g., monocytes/macrophages, microglia). But by an alternative “*trans-*signaling,” using the soluble IL-6 receptor (sIL-6R), it can exert its effect on many cell types including neurons (Zhou et al., 2016). The role of IL-6 in nervous tissue is “case-sensitive” as it was reported for the immune system: the classical signaling through mIL-6R initiates anti-inflammatory signals whereas the *trans-*signaling pathway is more pro-inflammatory (Rothaug et al., 2016).

GM-CSF is more known of its hemopoietic function as a colony stimulating factor of the granulocyte-macrophage lineage, but it is also a pro-inflammatory cytokine with numerous functions in innate and adaptive immunity (Donatien et al., 2018). GM-CSF was already shown to play role in tumor-nerve interaction in bone cancer pain (Schweizerhof et al., 2009).

And it was found to initiate central sensitization by inducing microglial BDNF secretion in chronic post ischemic pain (Tang et al., 2018). Besides microglia its receptor (GM-CSFR) is expressed on cortical and hippocampal neurons (Krieger et al., 2012).

The chemokine CCL5 (RANTES), as other members of the chemokine family regulate leukocyte trafficking to sites of inflammation and it is a dominantly pro-inflammatory agents.

CCL5 can bind to three receptors chemokine (C-C motif) receptor 1 (CCR1), CCR3 and CCR5 and is involved in several pain states (Yin et al., 2015). The main receptor, CCR5 is expressed by microglial cells and it induces the alternatively activated M2 phenotype differentiation (Laudati et al., 2017), which is known to inhibit pro-inflammatory signals. In this way, CCL5 may have an anti-inflammatory, anti-nociceptive role when it activates the CCR5 receptor.

The NF-kB pathway plays crucial role in the astrocyte specific response for IL-1β stimulation. Besides its important function during inflammation in peripheral tissues, it is also a mediator of cytokine and chemokine secretion by astrocytes. Genes encoding the three selected cytokines were shown earlier to be associated with the NF-kB signaling pathway in other cell types (Schreck and Bauerle, 1990; Wickremasinghe et al., 2004; Son et al., 2008).

In summary, the three of the selected cytokines/chemokines and also the NF-kB pathway can all be important target molecules in inflammatory pain conditions. Specific receptors of the three molecules were shown on microglial cells. GM-CSFR is expressed on neurons and for IL-6 *trans-*signaling is available for its neuronal effect. The astrocytic production of these molecules support the theory of the indirect action of IL-1β, by inducing astrocytic secretion of mediators which can be means of complicated networks of glia-neuron and glia-glia interactions and can contribute to the enhanced activity of the local cellular networks thus can be part of the process of central sensitization. Another interesting feature of this system is, that astrocytes themselves produce IL-1β and it would be important to understand the consequences of this “autocrine-loop” and possibly influence its functioning to shift astrocytic activation toward the A2 “protective-type” to attenuate the activity of the local cells which may lead to the inhibition of the pro-nociceptive processes.

## Data Availability Statement

The raw data supporting the conclusions of this manuscript will be made available by the authors, without undue reservation, to any qualified researcher.

## Ethics Statement

The animal study was reviewed and approved by Animal Care Committee of the University of Debrecen, Hungary according to national laws and European Union regulations [European Communities Council Directive of 24 November 1986 (86/609/EEC)].

## Author Contributions

KHo designed the experiments, analyzed data and prepared the manuscript. AG and KHe carried out the immunocytochemical stainings on the spinal cord sections. AG, KHe, and EB conducted the behavioral experiments and prepared the astrocyte cultures. LD performed the IMARIS analysis of the sections and reviewed the manuscript. AG photographed the immunostained sections. KHo and EB carried out the Proteome Profiler, ELISA and western blot experiments. All authors read and approved the manuscript.

## Conflict of Interest

The authors declare that the research was conducted in the absence of any commercial or financial relationships that could be construed as a potential conflict of interest.
