# Supplementary material for: IL-1β Induced Cytokine Expression by Spinal Astrocytes Can Play a Role in the Maintenance of Chronic Inflammatory Pain
Source: Front Physiol. 2020 Nov 16;11:543331. doi: 10.3389/fphys.2020.543331 (PMC7701125; doi:10.3389/fphys.2020.543331)

Supplementary material:summary of primary and secondary antibodies used in the study

PRIMARY ANTIBODIES USED IN THE STUDY			
**antigen**	host	dilution	manufacturer
**IL-6**	rabbit	1:500 WB and IHC<br>1:1000 ELISA	PeproTech
**GM-CSF**	rabbit	1:500 WB and IHC<br>1:1000 ELISA	PeproTech
**CCL5**	rabbit	1:500 WB and IHC<br>1:1000 ELISA	PeproTech
**NF-κB p65**	rabbit	1:1000	Invitrogen
**NF-κB p50**	mouse	1:500	Santa Cruz Biochemicals
**iκB**	mouse	1:250	Santa Cruz Biochemicals
**GFAP**	mouse	1:2000	Synaptic Systems
**β-tubulin**	mouse	1:2000	Sigma

SECONDARY ANTIBODIES USED IN THE STUDY			
**antibody**	conjugated	dilution	manufacturer
**goat-anti-rabbit IgGs**	HRP	1:1000	DAKO
**goat-anti-mouse IgGs**	HRP	1:1000	DAKO
**goat-anti-mouse IgG**	Alexa Fluor 488	1:2000	Invitrogen
**goat-anti-rabbit IgG**	Alexa Fluor 555	1:2000	Invitrogen
**goat-anti-rabbit IgGs**	biotin	1:200	Vector Labs

Supplementary material: antibody depletion

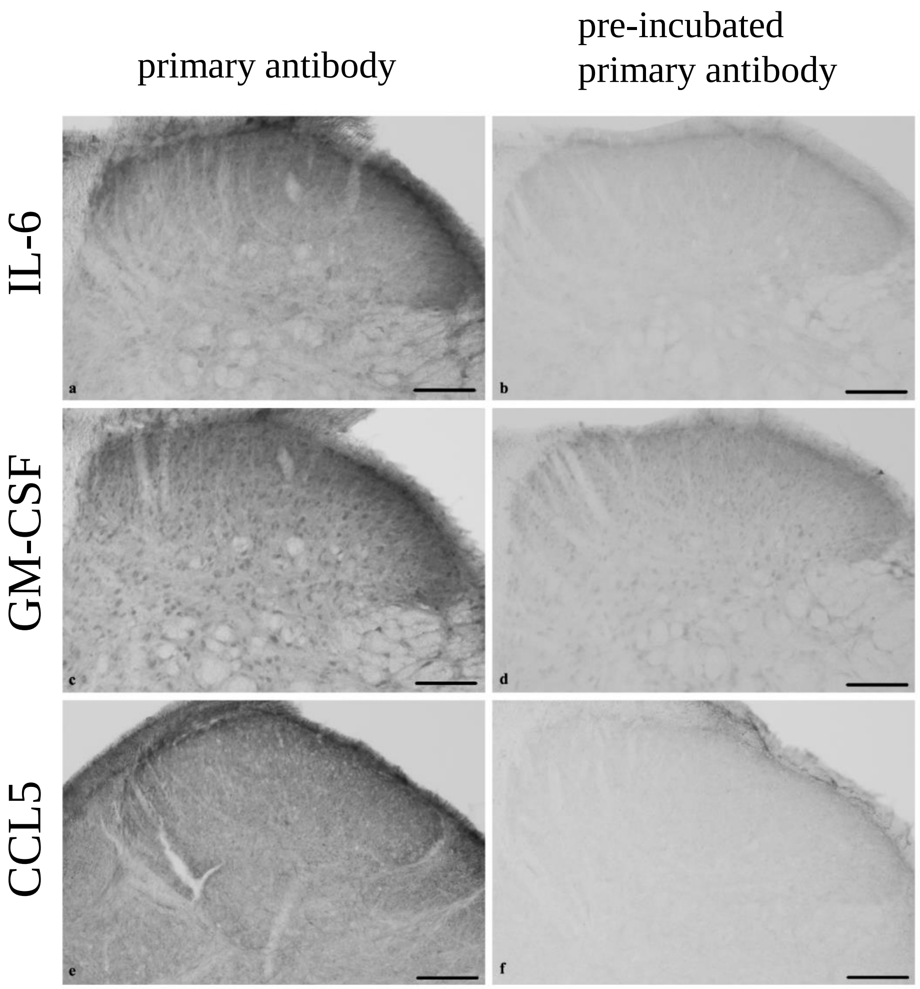

scale bar: 100 μm

Supplementary material: western blots

IL-6

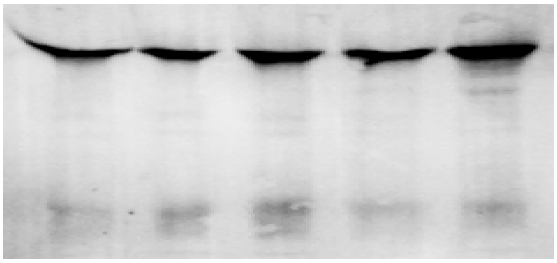

GM-CSF

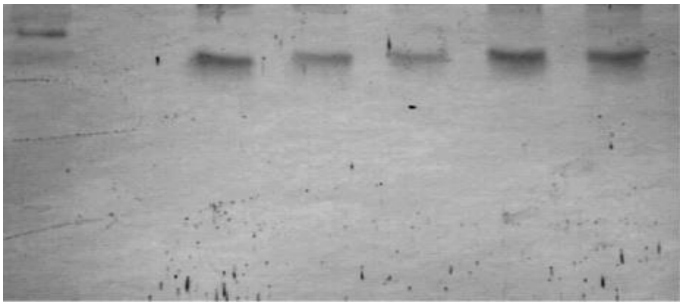

CCL5

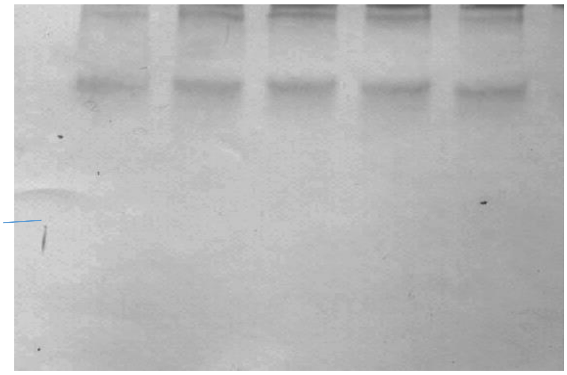

p65
cytosol

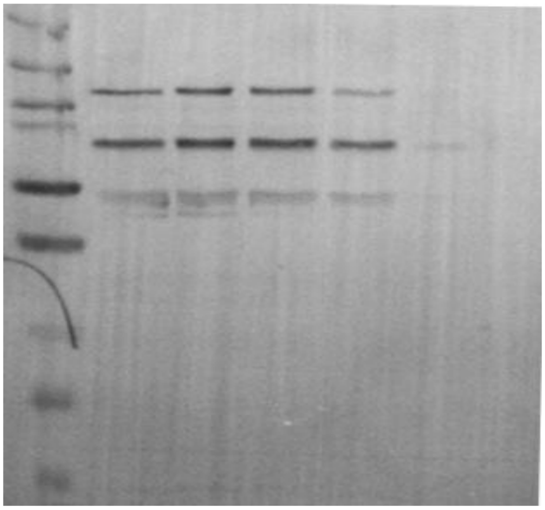

p65

B-tubulin

p65
nuclear

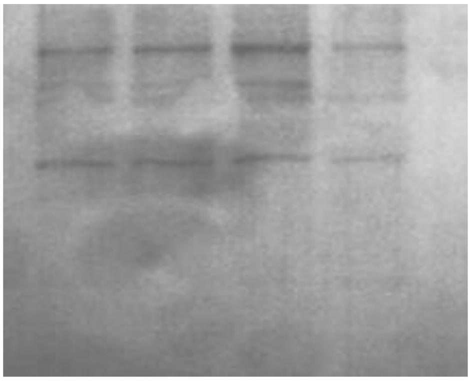

p65

PCNA

ikB

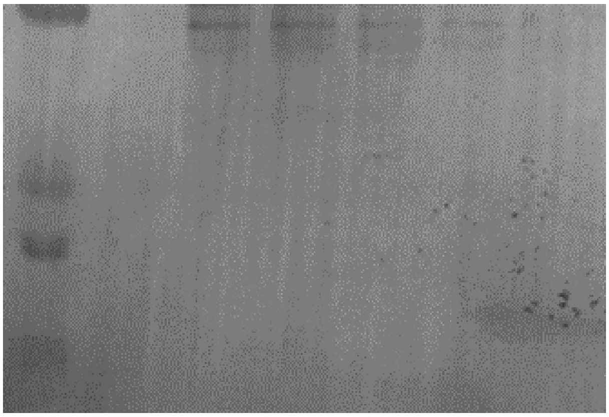

GFAP

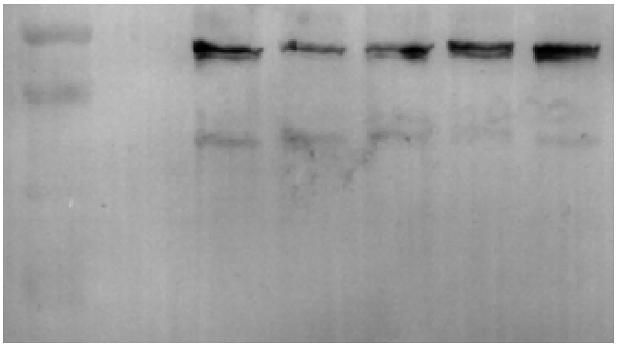

Supplement: Supplementary file 1 [file Data_Sheet_1.PDF]
