# Supplementary figures and images for: IL-1β Induced Cytokine Expression by Spinal Astrocytes Can Play a Role in the Maintenance of Chronic Inflammatory Pain
Source: Front Physiol. 2020 Nov 16;11:543331. doi: 10.3389/fphys.2020.543331 (PMC7701125; doi:10.3389/fphys.2020.543331)

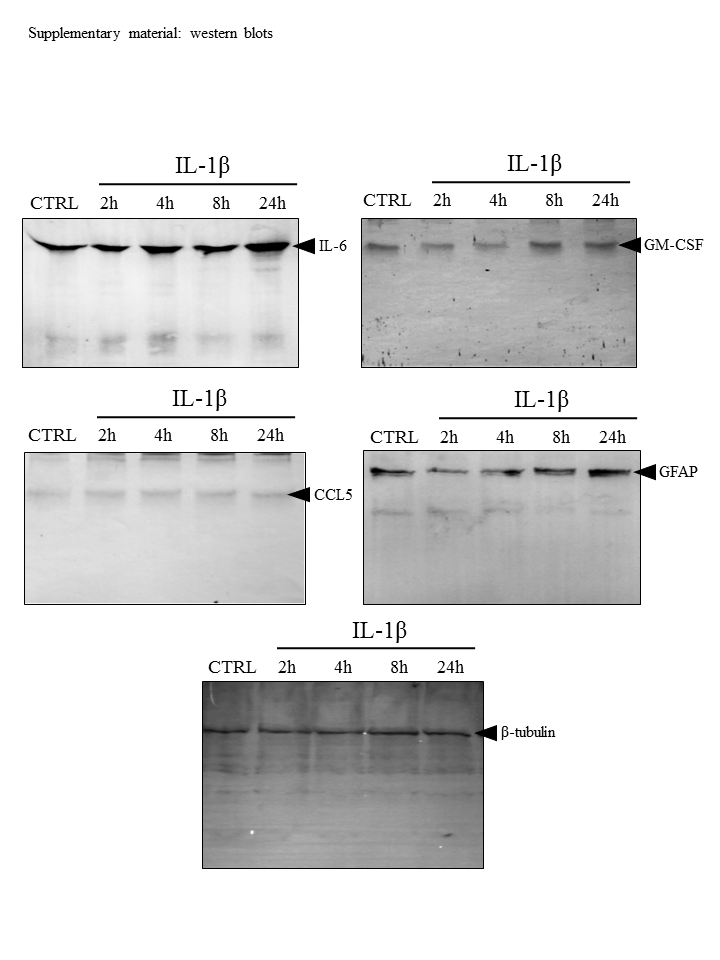

Supplement: Supplementary file 2 [file Image_1.tif]

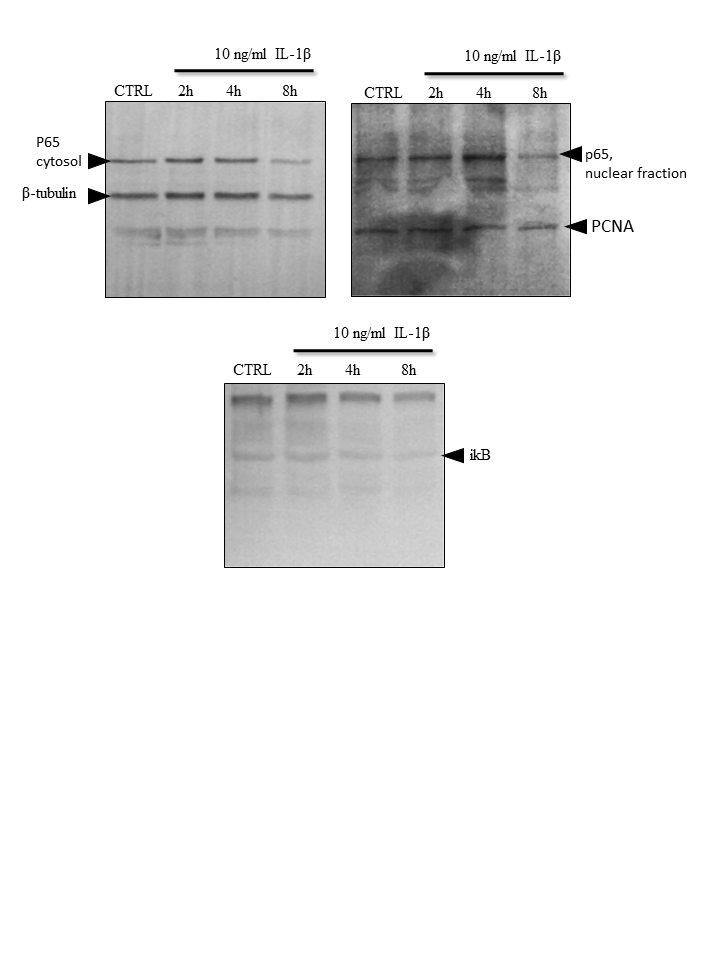

Supplement: Supplementary file 3 [file Image_2.tif]

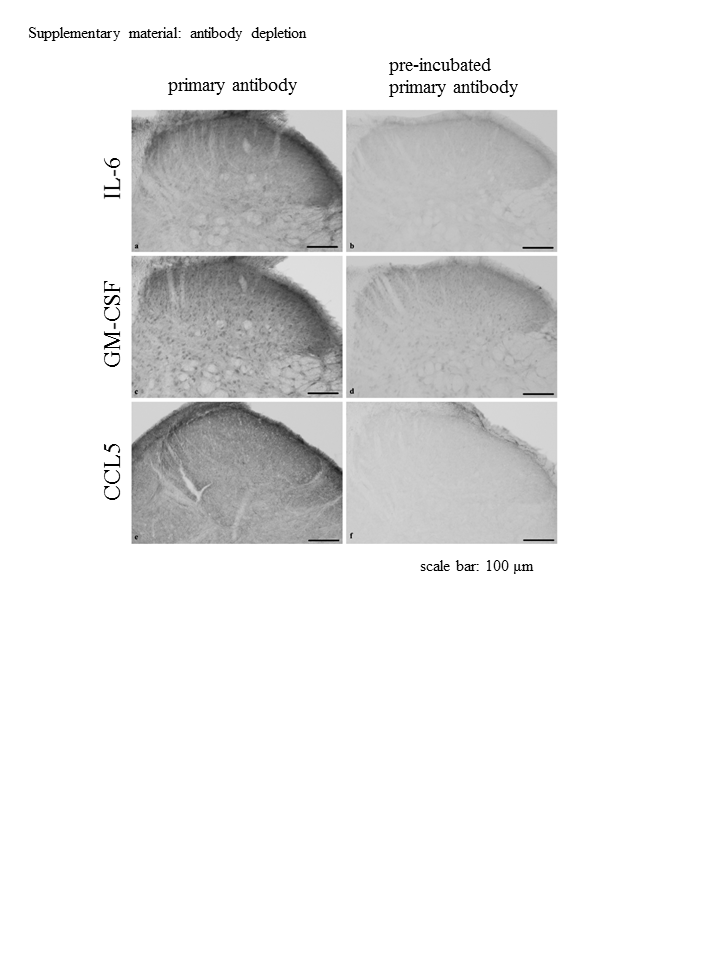

Supplement: Supplementary file 4 [file Image_3.TIF]
